# Acute Gastric Volvulus Secondary to Malrotation of Gut in a Child with Cerebral Palsy

**Published:** 2011-07-30

**Authors:** Kanchan Kayastha, Afzal Sheikh

**Affiliations:** Department of Pediatric Surgery, The Children's Hospital and the Institute of Child Health Lahore, Pakistan

**Keywords:** Acute gastric volvulus, Gastric ischemia, Malrotation, Gastropexy

## Abstract

Acute gastric volvulus secondary to malrotation of gut is a rare surgical emergency. We report a case of an eight years old cerebral palsy (CP) child who presented to us with sudden upper abdominal distension and non productive retching. X-ray abdomen revealed a huge gas shadow on left side of abdomen with paucity of distal gas shadows. On exploration organoaxial gastric volvulus with gastric ischemia, secondary to malrotation of gut, was found. Volvulus derotated and Ladd’s procedure was done. Gastropexy and fundoplication was not done due to gastric ischemia. Early diagnosis and surgical management can save the patient from fatal complications of gastric perforation due to gastric ischemia.

## INTRODUCTION

Gastric volvulus is an abnormal rotation of stomach, more than 180 degree, on its own axis. The predisposing factors of gastric volvulus are either abnormal laxity of various ligaments around stomach or other anatomical causes. Malrotation of gut is an extremely rare cause of secondary gastric volvulus in which partial duodenal obstruction leads to the distension of stomach followed by twisting. Gastric ischemia may occur due to strangulation of stomach [1, 2, 3].

We report a patient of cerebral palsy with acute gastric volvulus and ischemia secondary to the malrotation of gut.

## CASE REPORT

 An eight years old girl with cerebral palsy presented with 4-day history of abdominal distension and retching. On examination there was marked distension of left side of epigastrium. Superficial palpation revealed slight tenderness over the distended area. The child was in obvious discomfort. Her vitals were - temp 101 °F, BP 110/70, pulse 120/min, respiratory rate 30/min. She was mildly dehydrated. Intravenous fluids and antibiotics were started. Nasogastric tube was passed but it could not decompress the distension. Baseline investigations were done; blood picture revealed leukocytosis. X-ray abdomen was performed which showed a huge air fluid level on left side with paucity of distal gas shadows (Fig. 1).

**Figure:**
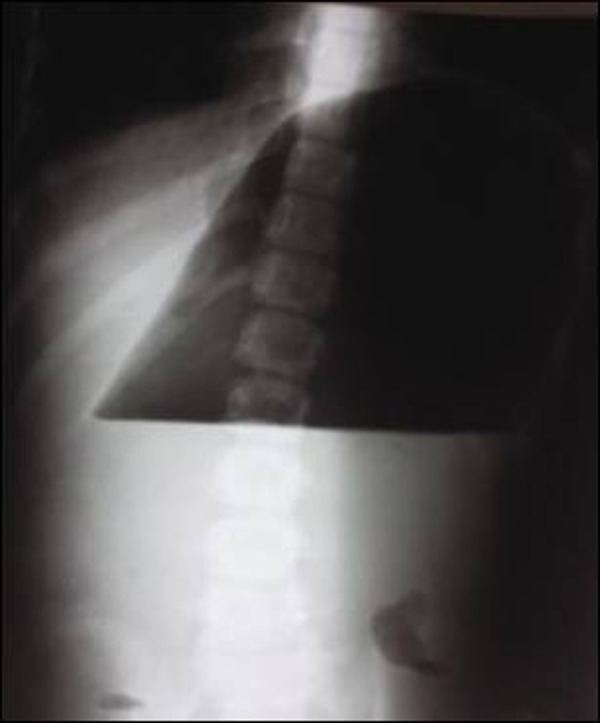
Figure 1: X-ray abdomen showing single air fluid level.

On the basis of the history, clinical examinations and x-ray abdomen, a provisional diagnosis of acute gastric volvulus was made. A decision for immediate exploration was taken. Blood was arranged and written consent was taken from parents for surgical exploration. Through supraumbilical transverse laparotomy incision a hugely distended stomach with greater curvature lying superiorly, was identified. The stomach was gently pulled out and derotated thus placing the greater curvature down to its original position. As soon as the stomach was placed in normal position the nasogastric aspirate increased and stomach got decompressed. There were ischemic patches over the anterior surface of stomach and the fundus which were left as such (Fig. 2, 3). On further exploration of abdomen malrotation was found with duodeno-jejunal junction ( DJ) on right of midline, mobile cecum and presence of Ladd’s band. The Ladd’s procedure was done to correct the malrotation. There was no diaphragmatic defect and spleen was placed in its normal position. Due to ischemia of the stomach fundoplication and gastropexy were not attempted.

**Figure:**
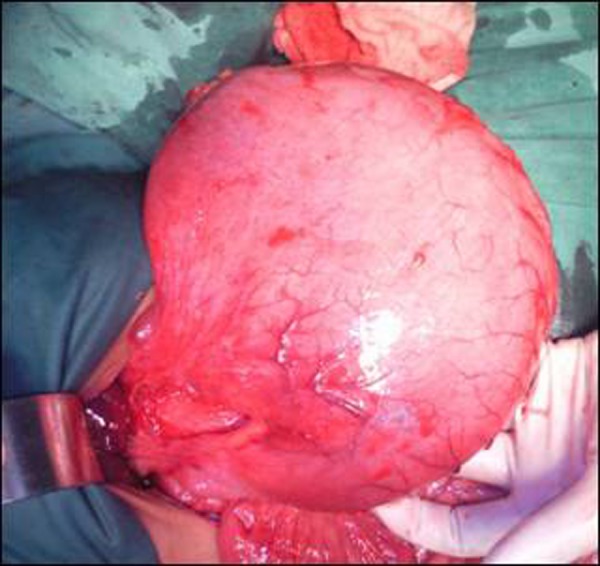
Figure 2: Hugely distended stomach retrieved out of abdomen.

**Figure:**
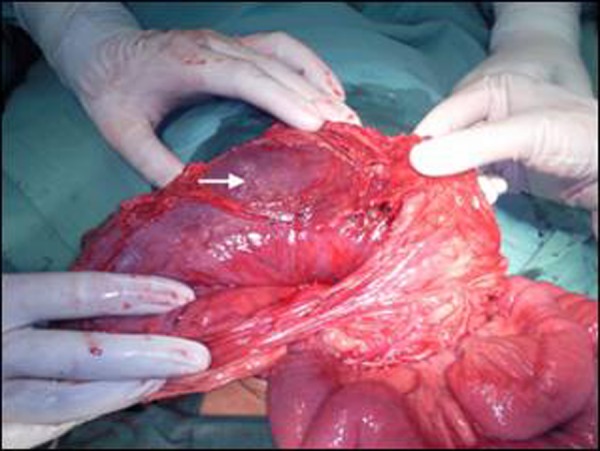
Figure 3: Showing ischemia of the stomach (Arrow).

The post-operative course remained uneventful and patient discharged on 8th day. In follow up she remained well and was referred to developmental OPD for management of cerebral palsy.

## DISCUSSION

 Gastric volvulus is a rare surgical emergency. According to the axis of rotation gastric volvulus can be organoaxial, mesentericoaxial or combined. In case of organoaxial volvulus the stomach rotates around the axis made by joining eosophagogastric junction and the pylorus. The greater curvature either lies anteriorly or superiorly, as found in our case [1].

The gastric volvulus can be primary which is the result of either absence or laxity of various ligaments attaching the stomach to the surrounding structures. The secondary gastric volvulus can occur due to various anatomic defects like diaphragmatic hernia, malrotation of gut, asplenia, wandering spleen, pyloric stenosis, traumatic injury to diaphragm, phrenic nerve palsy, abdominal tumors etc [3, 4, 5, 6, 7]. In our case, acute gastric volvulus was secondary to malrotation of gut. We hypothesize that partial duodenal obstruction might result in gastric over-distension that predispose to an abnormal rotation of stomach around its long axis.

The gastric volvulus may present as acute surgical emergency or with chronic intermittent symptoms. In acute gastric volvulus there is sudden upper abdominal distension associated with pain abdomen. Patient may present with retching as well. In adults the classical Borchardt’s triad may be found which include sudden pain in abdomen, non productive retching and inability to pass NG tube. In case of chronic gastric volvulus the abdominal pain and distension is intermittent as the stomach derotates itself at times. Early satiety and fullness after meal may be the symptoms [1, 3, 5].

Both plain and contrast radiographs of abdomen play vital role in diagnosis of gastric volvulus. Plain x-ray abdomen shows typical large air fluid level at the left upper quadrant with paucity of distal gas shadows in organoaxial rotation; similar was found in our case. The barium study is more specific as it can show the position of greater curvature, pylorus and the antrum if done with stomach in twisted state [3]. 

Acute gastric volvulus is a surgical emergency. The delay in the diagnosis and management may result in serious complications like gastric necrosis, gastric perforation, sepsis and cardiovascular failure [6]. In our case the gastric ischemia had occurred however the stomach was viable after derotation.

The surgical management of gastric volvulus is based upon three principles namely decompression of the distended stomach, correction of volvulus and prevention of recurrence. Both open as well as laparoscopic approaches have been advocated. Stomach should be inspected for any ischemia or necrosis due to the strangulation. If any doubt in viability of stomach arises, then segmental, subtotal or total gastrectomy can be done. Anterior gastropexy, gastrostomy or fundoplication may be added for prevention of gastric volvulus. However correction of various anatomical defects in case of secondary gastric volvulus should always be kept in mind [1, 2, 3, 4, 5]. Our patient was managed without any additional procedure on stomach as it was secondary to malrotation.

## Footnotes

**Source of Support:** Nil

**Conflict of Interest:** None declared
